# A qualitative study examining everyday frailty management strategies adopted by Polish stakeholders

**DOI:** 10.1080/13814788.2019.1668372

**Published:** 2019-10-07

**Authors:** Maria Magdalena Bujnowska-Fedak, Holly Gwyther, Katarzyna Szwamel, Barbara D’Avanzo, Carol Holland, Rachel L. Shaw, Donata Kurpas

**Affiliations:** a Department of Family Medicine, Wrocław Medical University, Wrocław, Poland;; b Centre for Ageing Research, Division of Health Research, Lancaster University, Lancaster, UK;; c Opole Medical School, Opole, Poland;; d Istituto di Ricerche Farmacologiche Mario Negri IRCCS, Milan, Italy;; e School of Life & Health Sciences, Aston University, Aston, UK

**Keywords:** Frailty, general practice, preventive medicine

## Abstract

**Background:** Frailty is a multidimensional clinical state that is common in older age and can be managed through intervention. Strategies to manage frailty have not been previously explored with stakeholders in Poland. This may stem from misperceptions about the nature and malleability of frailty, which has resulted in it being viewed as a lower priority healthcare concern.

**Objectives:** To explore stakeholders’ views to determine whether there are effective everyday strategies that they can adopt to reduce, reverse or prevent frailty.

**Methods:** Semi-structured focus groups were conducted with five stakeholder groups (frail/pre-frail and robust older adults, health and social care professionals and family caregivers) in Poland (*n* = 44). Data was analysed using a reflexive thematic analysis approach.

**Results:** Two themes were developed. The first emphasized both the positive everyday and more effortful strategies used by individuals to counter frailty; these included the adoption of healthy lifestyle behaviours, social engagement and shared experiences. Stakeholders perceived that older adults, even frail ones, might benefit from engaging in meaningful activities to build resilience against frailty. The second examined formal interventions delivered by health and social care professionals. Stakeholders noted the need to increase awareness of the malleability of frailty among professionals.

**Conclusion:** Raising awareness of the malleability of frailty amongst health and social care professionals is critical. Further, information provision and personal support should be essential elements of health interventions aimed at older adults and family caregivers. Interventions to support resilience building in older adults should also be framed within a model of fostering self-efficacy.

KEY MESSAGES

- Stakeholders suggested that frailty should be viewed as a dynamic process with opportunities for treatment and improvement.
- Stakeholders believed that even frail older adults would benefit from engaging in meaningful but everyday activities to build resilience against frailty.
- Interventions designed to manage frailty should be carefully labelled to avoid stigma.

## Introduction

Frailty is a multidimensional, clinical condition characterized as a state of increased vulnerability to adverse health outcomes when exposed to a stressor, for example, a chronic disease diagnosis, an acute infection, or a fall [1–3]. Frailty becomes increasingly common at older ages [4]. However, it is not an inevitable part of ageing. Evidence suggests that treating frailty in older adults is a realistic therapeutic goal [5], and as a dynamic state characterized by modifiable transitional stages [6,7], it can be improved through intervention [8–10].

There are many modifiable physical, psychological and social factors including obesity [11], malnutrition [12], vitamin D deficiency [13], a sedentary lifestyle [14], loneliness and a lack of involvement in social activities that predispose older adults to frailty [15,16]. These factors provide ideal targets for interventions designed to reduce, reverse or prevent frailty in older adults. Despite recent evidence for the effectiveness of interventions for frailty [17], awareness around the prevention and management of frailty among health and social care professionals across Europe is limited [18].

To engage in preventative strategies, older adults, their families and associated professionals need first to understand the dynamic and malleable nature of frailty and have confidence in the possibility of reversing its effects and maintaining a good quality of life [9,10,18]. Although a number of interventions designed to manage frailty have demonstrated success, particularly exercise or multicomponent interventions in a group setting [8,9], these are often localized, formalized and short-term interventions in a specific research or healthcare setting.

Previous work with European stakeholders [18], including Polish nationals, raised awareness of the lack of understanding around the transitional nature of frailty and the cultural challenges associated with delivering interventions in the complex Polish health and social care system. As is typical in much of Central and Eastern Europe, in Poland, health and social care is deeply fragmented and there is a view that the disparate systems are underequipped to meet the needs of older adults [19]. Furthermore, there appears to be a cultural preference for formal health and social care interventions led by professionals [18]. Such interventions may put additional pressure on an already burdened system. Therefore, this study aims to explore the views of key stakeholders to determine whether there are effective everyday strategies that stakeholders can adopt to reduce, reverse, or prevent frailty.

## Methods

This study forms part of an analysis of stakeholders’ needs which was conducted as part of a larger European Union-funded project (FOCUS 664367) [20,21]. The data collection methods have been reported previously [18,19] but are repeated briefly here.

### Ethics and consent to participate

The research was approved by the Bioethics Commission of Wrocław Medical University; approval no. KB-502/2015. All participants gave written informed consent.

### Participants

Focus groups were conducted with five stakeholder groups—healthy adults, frail older adults, healthcare professionals, social care professionals, and family caregivers. Recruitment strategies have been described previously but in brief [18], older adults (over 65 years) and caregivers were recruited purposively from general practice (GP) clinics across an urban Lower Silesia District of Poland through advertising given during an appointment. Participant information sheets were given to older adults that stated: ‘we are interested in hearing from people who consider themselves frail or infirm as well as people who regard themselves as healthy and active.’ People were invited only to participate if they wished to do so. Older adults self-identified as frail, a view which was confirmed by a physician’s clinical judgement. Individuals with severe dementia and/or terminal illnesses were excluded. Caregivers were contacted through health and social care services. They were required to be taking care of a frail older adult regularly, but were not necessarily co-resident. Healthcare professionals from the same region were contacted through professional networking (in person, by telephone, and via email) and were required to have an active role in either geriatric inpatient or outpatient services and at least two years’ experience in their field. Social care providers were recruited through social care services in the region and were similarly required to have two years’ experience. Social care professionals were given time away from work to participate.

### Data collection

Focus groups were conducted between October 2015 and January 2016. The purpose of the study was described at the start of each focus group, which was done separately for each stakeholder group and lasted 48–90 min. Focus groups were facilitated by two female GPs (DK and MBF) with limited experience of qualitative research. Researchers were not known to the participants and no personal information was provided. Discussions with older adults and caregivers were held near the residence of the older participants, mainly in the educational centre of Wrocław Medical University. Health professionals met in the seminar room at the university, and social care professionals’ discussions took place in a regional welfare centre.

Semi-structured questions encompassing views on frailty and experiences and expectations of health and social care were defined in advance by the authors and based on the available literature. Interview questions have been previously published [18].

### Data analysis

Discussions were digitally recorded and transcribed verbatim. Transcriptions were independently analysed by MBF, DK and KS using an inductive reflexive thematic analysis approach [22], which is flexible in that it is not tied to any epistemological or theoretical perspective. Codes were manually assigned, collated and compared within and across transcripts. The initial themes and salient quotations were translated into English and developed through further interpretative work by HG. Supplementary discussions with RS, BDA and CH developed the themes still further. Validity and reliability were protected through constant exchange between the authors.

For transparency, the authors do not claim that saturation was achieved, in terms of reaching the point at which no new codes could be generated [23]. Rather, we are confident that the sampling strategy was realistic, appropriate, and adequate for the research question and study design. The methods used in this study consider qualitative research reporting guidelines [24].

## Results

Participants’ (*n* = 44) characteristics can be found in Tables 1–3. Their names and personal information have been anonymized. Two themes were developed and are illustrated by representative quotations in Tables 4 and 5.

**Table 1.** Older adults.

Stakeholder group	Age range	Sex	Living arrangements	Number of chronic diseases
Frail and pre-frail older adults (FOA)	73–89	Female	With family	3
		Male	With spouse	7
		Male	With spouse	6
		Female	With spouse	6
		Male	With spouse	8
		Female	With spouse	7
		Female	With spouse	6
		Female	Alone	7
		Female	With family	9
Healthy older adults (HOA)	68–89	Female	Alone	2
		Male	With spouse	2
		Female	Alone	5
		Male	With spouse	3
		Male	With spouse	2
		Female	Alone	3
		Male	With spouse	5
		Female	With spouse	3
		Female	With spouse	4
		Male	With spouse	6
		Male	Alone	2

**Table 2.** Family caregivers.

Stakeholder group	Age range	Sex	Relationship with older adult	Caregiving experience (years)
Family caregivers (FC)	61–77	Female	Adult offspring	12
		Female	Adult offspring	5
		Female	Adult offspring	10
		Female	Adult offspring	3
		Male	Adult offspring	10
		Male	Spouse	3

**Table 3.** Professional stakeholders.

Stakeholder group	Age range	Sex	Profession	Experience (years)
Healthcare professionals (HCP)	31–58	Female	Physician	12
		Female	Physician	21
		Male	Physician	23
		Female	Physician	16
		Female	Physician	24
		Female	Physician	3
		Female	Nurse	30
		Female	Nurse	25
		Female	Nurse	7
Social care professionals (SCP)	43–64	Female	Social care worker	2
		Female	Social care worker	16
		Female	Social care worker	33
		Female	Social care worker	34
		Female	Social care worker	10
		Male	Social care worker	20
		Male	Social care worker	31
		Female	Social care worker	18
		Female	Social care worker	15

**Table 4.** Representative quotations for theme 1: Personal strategies to build resilience.

Theme 1	Key concepts within theme	Example quotations
Personal engagement and strategies to build resilience	Preserving physical identity/capability	*Most importantly, I want to be healthy. This is the basic thing, wanting to stay healthy. Of course, there are various ailments. I'm not saying there aren't. […] So I'd say that I don't always feel like running, but I know I'm running to stay healthy. Who else is supposed to make me healthy? I'm sorry, but medicine won't give me health*. [HOA9]
		*I do different things […], of course, physical exercises, riding a stationary bicycle, or walking more often, then you immediately feel better, you can feel completely different*. [FOA3]
		*Physical activities at home, performance of all household chores, I do as much as I can. I think it is important*. [HOA6]
	Preserving social identity and sense of purpose	*The dog keeps me connected with people. And even when I feel down or in a terrible mood, and someone says something, and I smile… “Good morning, how are you?” and I feel better. I’ve met many friends that way*. [HOA8]
		*If it wasn't for my dog, I wouldn't have got up in the morning but it used to come and whimper. I had to get up and walk five kilometres*. [HOA5]
		*My observations are that the family members are often overprotective and do everything for the patient, including changing the position on the bed, shaving, even in rehabilitation wards where I have had an opportunity to work, the patient is no longer able to take care of himself/herself, but the worst cases are the wives who do everything for their husbands: sit down, I'll undress you, I'll put all the things in the cabinet*. [HCP2]
		*I have a grandmother who is 90 years old […]. She is ailing, frail, she lives her life through her children, grandchildren, great-grandchildren. She makes jam, she can't walk but her hands are good, she tries very hard to stay well, even though she has pulmonary embolism and other serious diseases. She absolutely refuses to give up*. [HCP2]
		*We have older volunteers for older people, they are women of around 60 years of age and they provide voluntary services to the older people with our help. We had evaluation meetings with these volunteers, they are over the moon, satisfied, they say they feel needed*. [SCP5]
		*Obviously, for an older person, [meeting a volunteer] is thrilling, they must get ready, […]. After all, somebody's coming, you need to clean up a little, think about having a guest*. [SCP5]

**Table 5.** Representative quotations for theme 2: Organizational strategies to build resilience.

Theme 2	Key concepts within theme	Example quotations
Organizational strategies to build resilience	Raise awareness of frailty as a medical condition	*Doctors diagnose the diseases that they know. When they do not understand a specific illness, they will not recognize it. Then there is no way of starting treatment or making the environment right for the patient, or making his/her family focus on the right issues*. [HCP6]
		*I think we should start by raising awareness among both doctors and families that it might be old age, but there is something we can do about it*. [HCP5]
	Screen and monitor for frailty to provide timely treatment	*We've known these patients for a dozen years or more, we work at the same place […] It creates complacency […] I see a record on my desk and before the patient comes in, I already know what the problem is. And at this point I think that such a survey tool, just to be able to look at it through some objective tool from time to time […] Could also be useful*. [HCP8]
		*If we involve them in the screenings, I think that 90, perhaps 80% of them will be very pleased; they will feel like they are being taken care of. And through the same activities, tests, consultations, education, I think we can improve the quality of life of these older patients to a certain degree and reduce the symptoms of frailty*. [HCP7]
	Provide information, support and advice	*The nurse even told me […] that I had to take a urine sample from my mum. She told me that I obviously needed a referral. It goes without saying, but you must get all the equipment. […]. So I'm supposed to buy all the equipment. I'm not sure it's okay. I have no idea what kind of equipment it's supposed to be [FC6] I also wouldn't know*. [FC2]
		*There isn't enough information; what we can and can't do, what we should and shouldn't do*. [FC2]
		*And we […] would go to a senior club and speak about what our MOPS [Municipal Social Welfare Centre] had to offer, about all our services*. [SCP5]
	Provide integrated services	*First a meeting, and then additional [printed materials] so that I can review them at home*. [FC4]
		*I would like to meet with the social care from my area, with nurses and doctors working in the field. […] Everybody will say how they see things, what problems they face, what's important in their job, and then we can look for a common ground*. [HCP6]

### Personal engagement and strategies to prevent or reverse frailty

This theme examined the everyday strategies used by individuals to manage frailty, according to their perceptions of how frailty was generated. Most of the stakeholders understood frailty as an end-of-life state rather than a condition that could be, to some extent, reversed or managed. Frail older adults focused on the physical difficulties encountered, including a gradual age-related decline in physical health and tiredness. Caregivers viewed frailty as a slowly developing, cumulative physical process but also noted that it was something that could suddenly become apparent, for example, after a fall. Professional stakeholders focused on how frailty was generated through psychological hardships, particularly social isolation and loneliness. They spoke about how physical frailty is closely related to, and even initiated by, a lack of mental wellbeing. Further, they suggested that frailty was not caused by chronic diseases per se, but rather by the psychological ability, or inability, to cope with illness; that is, a person’s individual resilience. Thus, they emphasized the idea that even when people presented with similar physical health concerns, their psychological response may be different and so they may need different levels of support to achieve the same health outcome.

### Preserving physical identity or capabilities

Stakeholders explored the significance of maintaining their physical, mental, professional, and social identities as well as ensuring a sense of purpose, or meaningful activity in their daily lives, even in circumstances of extreme frailty. Many of the physical activities described by older participants were normal, often solitary everyday activities, such as carrying out daily household chores and exercising but participants viewed them as critical in their self-care and they were consciously and intentionally carried out to prevent frailty. Other activities required more effort, for example, training to run long distances, while yet others had the additional benefit of providing an opportunity to socialize. These included tasks such as walking a dog or working on an allotment. Participants noted how having a pet gave them a purpose, motivated them to exercise and provided them with social interaction, which elevated their mood.

### Preserving mental and professional identity or capabilities

Preservation of mental identity was also critical; respondents stressed the importance of engaging in challenging cognitive tasks, such as memory exercises and everyday activities like reading books and using the Internet. Participants reflected that mental health was perhaps more critical in managing frailty than physical health. Some participants actively focused on maintaining their professional identity as a means of occupying their mind to overcome a possible alternate reality of a preoccupation with health or illness.

There was a view that maintaining health and preventing frailty was effortful and that people needed to ‘try-hard’ [HCP2] to stay well and avoid becoming frail. Participants described how health and social care professionals, and family caregivers could be ‘overprotective’ [HCP2] of frail older adults. Overprotectiveness arises from feelings of concern about the health of the frail person, and the view that doing too much would be harmful to them. However, some professionals suggested that frail older adults should undertake ordinary meaningful activities within their limitations and that preventing them from participating in regular activities like shaving or making jam, would expedite the frailty process.

### Preserving social identity and a sense of purpose

Membership of more formal groups including pensioners’ clubs, the University of the Third Age and volunteering schemes were also viewed as an effective means of preventing frailty. Stakeholders described the mutual benefits of volunteering, with opportunities to interact, as well as achieving a sense of purpose. As well as being inclusive for people with different needs, participants reported that these schemes provided a space for people to engage socially, often at low cost. However, these activities were noted as more effortful for some.

### Organizational strategies to prevent or reverse frailty

Stakeholders described a range of existing or desired formalized services and strategies to assist in managing frailty (Table 5).

### Raise awareness of frailty as a medical condition

There was a lack of recognition of frailty as a medical condition among some healthcare professionals. GPs acknowledged that to some extent, they did not consider frailty as ‘a health disorder’ [HCP1], rather a natural product of ageing. Thus, there was a view that it would be difficult to treat. Some stakeholders suggested that an awareness-raising campaign should be encouraged to educate people in the potential benefits of interventions for frailty prevention and management, perhaps in the first instance through a training intervention for healthcare professionals.

### Screen and monitor for frailty to provide timely treatment

Participants suggested that frailty screening may be useful, and might lead to the optimization of treatments, for example, through the reduction of unnecessary medications, and providing adequate instrumental support, including the provision of hearing aids, glasses, walking frames, and crutches. Stakeholders also described that regular screening might satisfy older adults and caregivers’ need for a connection with professionals, which would, in turn, build trust and enable the type of enduring healthcare relationships they preferred. GPs suggested that a screening tool would provide an objective rationale for treatment, thus reducing the likelihood of making subjective assumptions about certain patients.

### Provide information, support and advice

There were calls for additional support and education. Family caregivers, who were not medically trained, requested educational interventions in the form of advice and leaflets; specifically, they suggested that practical tips on how to administer medication or personal care services would be helpful. They also indicated that training or assistance could be given when novel or new needs arose, for example, in assisting with physiotherapy exercises, or medical procedures. There was also a need to ensure that training could be repeated or accessed promptly and that contacts with professionals and peer caregivers could be maintained over time.

### Provide integrated services

Some stakeholders described informal systems of information provision by councils to relevant groups, for example, through expert speakers attending community meetings. While this was recognized as beneficial, and as an opportunity to socialize and share experiences, stakeholders expressed a need for more formal and specific meetings with and between health and social care professionals. According to social care professionals, care is disjointed and there are limited opportunities to exchange views with their medical counterparts. Social care professionals suggested that frequent multidisciplinary meetings could benefit everyone, in terms of knowledge exchange and providing a more integrated and person-centred care for frail older adults.

## Discussion

### Main findings

This study highlights the need to raise awareness of the malleability of frailty amongst health and social care professionals, as well as noting a number of strategies that older adults and their caregivers can adopt to reduce, reverse or prevent frailty in their everyday lives. These strategies include engagement in meaningful physical, psychological, social and even professional activities.

### Interpretation in relation to the existing literature

Social contact was critical to all the stakeholders in preventing or reducing frailty. There was a view that having a purpose in life, feeling useful, and remaining socially active may lead to fewer medical interventions. These findings support the work of Tanaka et al. [25], who concluded that early interventions for social frailty might prevent physical frailty in community settings. The importance of maintaining or enabling social relationships was similarly a key factor in other studies conducted as part of the FOCUS project. Social connectedness appears to improve adherence to interventions for frailty [17] and enables people to maintain a sense of self and build resilience in adversity [18]. Although some of these strategies were relatively informal, others require a higher level of organization and delivery, and so may not be practicable for all.

The provision of psychological support may also be relevant in building resilience against frailty. Some of the older adults in this study were highly motivated individuals with a keen interest in, and control over their physical health. However, not everyone possesses such a strong sense of self-efficacy, and this should be explored in future studies. It may be that self-efficacy can be developed through mixing with others and seeing ‘other people like me’ being successful in managing their frailty. This, in turn, may help generate an individual’s confidence to do something about their health status. However, physical activity should be personalized preventing people from becoming discouraged [26]. Thus, interventions to support resilience building in older adults should be framed within a model of fostering self-efficacy.

Individual stakeholder groups expressed their willingness to participate in local group meetings, specifically to promote care coordination between the professionals and to engage socially with others. The impact of this on the development of frailty is threefold. Firstly, it may be that through active engagement in comparisons of shared experiences, individuals and caregivers realize that frailty is developing and can then go on to access treatment. Secondly, people would have access to social contact, a cornerstone in frailty prevention. Thirdly, facilitating these meetings may also provide opportunities to implement health promotion interventions, such as raising awareness of the reversibility of frailty. Such knowledge could shift conceptions of frailty and, as a result, change the way frailty is managed in the field of health behaviour.

Given that the term ‘frailty’ has negative connotations [27], we suggest that interventions to support frail older adults are conceptualized differently in terms of their language and focus on ‘building resilience’ rather than preventing or reversing frailty as this may make them more acceptable to older adults. Understanding frailty as a loss of resilience, with the ensuing opportunity to rebuild resilience in one of the key frailty deficit areas, perhaps in terms of improving physical health, cognitive health issues or social connectedness, may mean that people are more likely to take action, than if they perceive pre-frailty and frailty as an end-of-life state.

Frailty is a complex phenomenon, but should be viewed as a dynamic process with opportunities for treatment. It is also suggested that interventions are relabelled as ‘resilience building’ to avoid any stigma associated with frailty and framed within a model of self-efficacy. Future research should explore the most effective ways of increasing awareness around the malleability of frailty in health and social care professionals while avoiding stigmatizing frail older adults. This would also fit with the aims of the ADVANTAGE European Joint Action on frailty (http://www.advantageja.eu/). Future research is also needed to explore the best ways of supporting personal resilience and coping strategies for those who may be feeling overwhelmed by the accumulation of difficulties they are facing. Similarly, work should be carried out with older adults to develop greater resilience within their social relationships, and to encourage health and social care professionals to move away from an entire deficit model and instead to take a more appreciative and positive approach to interventions with older adults that focus on living well in older age.

### Strengths and limitations

Older adults self-identified as frail in the absence of a standardized measure at the time of the study. Currently, there is a range of studies investigating the best tool to use to measure frailty in various circumstances. With the benefit of recent research [28], an accumulation of deficits model, or frailty index would have been preferable to self-identification [29]. Irrespective, this issue only affects one of the groups of stakeholders (*n* = 9) and given that the ‘frail’ label is generally resisted by older adults [30], it is reasonable to assume that those people who identified as frail were indeed so.

The GPs conducting the focus groups had limited experience of qualitative research; however, they were supported by the wider authorship team, some of whom have extensive experience of qualitative research. Although these results originate from a purposive study of Polish stakeholders, there are transferable lessons to other Central European and wider European healthcare systems, including the need to generate awareness of the malleability of frailty, to develop interventions that build self-efficacy and resilience in older adults and their caregivers, and the need for integrated working between health and social care professionals.

## Conclusion

Our study indicated that stakeholders believe that older adults, even particularly frail ones, could benefit from engaging in meaningful everyday activities to build resilience against frailty. Self-efficacy was identified as a facilitator of resilience-building activities and overprotectiveness of health and social care staff was identified as a barrier. A lack of awareness around the malleability of frailty was noted amongst health and social care professionals which suggest a need for future training. A more positive approach to interventions for frailty, as well as additional information, support and advice for caregivers may be beneficial.
